# Benzene and its metabolite decreases cell proliferation via LncRNA-OBFC2A-mediated anti-proliferation effect involving NOTCH1 and KLF15

**DOI:** 10.18632/oncotarget.16588

**Published:** 2017-03-28

**Authors:** Pengling Sun, Jing Wang, Xiaoli Guo, Yujiao Chen, Caihong Xing, Ai Gao

**Affiliations:** ^1^ Department of Occupational Health and Environmental Health, School of Public Health, Capital Medical University, Beijing, China; ^2^ Beijing Key Laboratory of Environmental Toxicology, Capital Medical University, Beijing, China; ^3^ Key Laboratory of Chemical Safety and Health, National Institute for Occupational Health and Poison Control, Chinese Center for Disease Control and Prevention, Beijing, China

**Keywords:** 1, 4-Benzoquinone, lncRNA, cell proliferation, RNA fluorescence in situ Hybridization assay

## Abstract

LncRNA has been considered to play a crucial role in the progression of several diseases by affecting cell proliferation. However, its role in benzene toxicity remains unclear. Our study showed that the expression of lncRNA-OBFC2A increased accompanied with the change of cell proliferation related-genes in benzene-exposed workers. *In vitro* experiments, 1,4-Benzoquinone dose-dependently inhibited cell proliferation and simultaneously caused the decrease of NOTCH1 expression and the increase of KLF15 in AHH-1 cell lines. Meanwhile, 1, 4-Benzoquinone obviously increased the expression of lncRNA-OBFC2A, which was consistent with our previous population results. Therefore, we propose that lncRNA-OBFC2A is involved in benzene toxicity by regulating cell proliferation. Further, we successfully constructed a lentivirus model of interfering the expression of lncRNA-OBFC2A. After interfering lncRNA-OBFC2A, the cell proliferation inhibition and the expression of NOTCH1 and KLF15 induced by 1, 4-Benzoquinone were reversed. Subsequently, RNA fluorescence in situ Hybridization assay showed that lncRNA-OBFC2A was located in cell nuclei. These results suggest that benzene and its metabolite decreases cell proliferation via LncRNA-OBFC2A-mediated anti-proliferation effect involving NOTCH1 and KLF15. LncRNA-OBFC2A can be a potential biomarker for benzene toxicity.

## INTRODUCTION

Benzene, a significant organic solvent and industrial raw materials, widely exists in the production environment and living environment, especially in developing countries [1]. Currently, workers exposed to benzene in China are more than 50 million. People may arise dizziness, nausea, coma, organ (e.g., liver and kidney) failure, or an occurrence of blood diseases if an excessive amount of benzene is inhaled over a short period [2, 3]. Long term exposure of benzene can cause chronic benzene poisoning, damage to the human hematopoietic system, or even hematologic malignancies such as leukopenia, regeneration barrier anemia and leukemia [4–11]. In 1982, IARC formally designated benzene to be a human carcinogen. Although many domestic and foreign scholars have made a lot of research, but the mechanism of benzene poisoning is not completely clear. Meanwhile, there is no effective treatment on benzene poisoning. At present, there is still lack of a useful marker for benzene health care and risk screening.

In recent years, more and more non-coding RNA (ncRNA) have been indicated to play an important role in the occurrence and development of many complex diseases [12–18]. And lncRNAs are used as markers of many diseases [19]. For example, the abnormal expression of lncRNA-MALAT1 is seemed as a new biomarker of cadmium toxicity [20]. Furthermore, lncRNA-MALAT1 has been used as a marker to detect prostate cancer [21] and an application of lncRNA-MALAT1 as a marker of blood molecular markers for disease diagnosis [22]. All these evidences suggest that lncRNAs may be a potential marker in many processes of diseases.

Growing studies have shown that many lncRNA molecules are associated with the leukemia [23]. High expression of lncRNA-DLEU1 and DLEU2 is associated with chronic leukemia [24]. Inhibiting the expression of T-ALL-R-LncR1 can be used in the treatment of acute lymphoblastic leukemia [25]. Our previous research has found that lncRNA-OBFC2A was closely associated with chronic benzene poisoning [26]. However, the role and mechanism of lncRNA-OBFC2A in the benzene toxicity has not been clear.

Limited studies revealed that lncRNAs played a vital role in cell proliferation. For example, lncRNA-SPRY4-IT1 regulated the proliferation and differentiation of melanoma cells [27]. The expression of lncRNA-URHC had a significant promoting effect on the proliferation of HCC cells [28]. Knocking-down of lncRNA-H19 suppressed cell proliferation of human cancer cells [29]. LncRNA-NALT promoted cell proliferation of T ALL cells, and lncRNA-NALT was located in the upstream of NOTCH1 [30]. At the same time, NOTCH1 is a gene which can promote cell proliferation [31], and KLF15 negatively regulates estrogen-induced epithelial cell proliferation by inhibiting DNA replication licensing [32]. Therefore, we hypothesized that lncRNA-OBFC2A might be involved in benzene toxicity by regulating cell proliferation.

In this study, in order to investigate the role and mechanism of lncRNA-OBFC2A in the toxicity of benzene, we first observed the expression of lncRNA-OBFC2A and proliferation related genes in benzene-exposed workers. Then we used a series of detecting measures to explore whether lncRNA-OBFC2A was involved in benzene toxicity by constructing the lentiviral interference model and RNA-FISH assay in AHH-1 cells treated with benzene final metabolites 1, 4-BQ.

## RESULTS

### The expression of lncRNA-OBFC2A increased and was related to basic blood indicators in benzene-exposed workers

**Figure 1:**
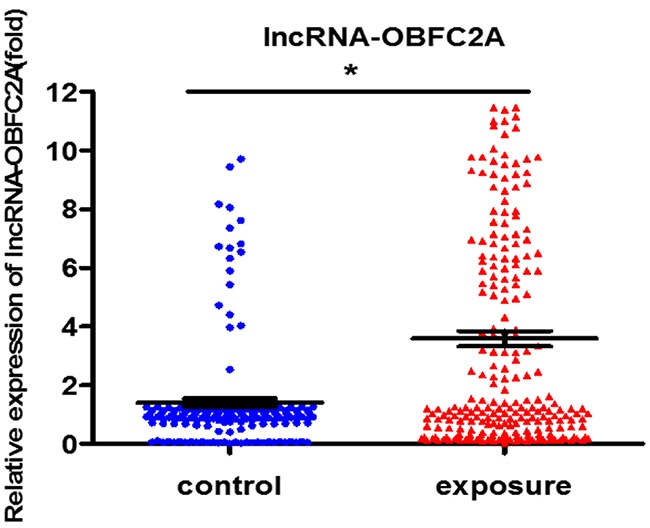
LncRNA-OBFC2A expression increased in benzene-exposed workers The expression of lncRNA-OBFC2A was detected by qRT-PCR. *P<0.05, compared with control. Data are expressed as mean ± S.D.; N (control)=170, N(exposure) =247.

In order to explore the toxicity of benzene, we detected the expression of lncRNA-OBFC2A and analyzed the correlation between lncRNA-OBFC2A and routine blood test in benzene-exposed workers. As shown in Figure 1, lncRNA-OBFC2A expression was obviously increased in workers exposed to benzene. And there was a close relationship between lncRNA-OBFC2A expression and basic blood indicators, further, the lncRNA-OBFC2A expression was significantly negative correlation with RBC (P<0.05) and HGB (P<0.05) (Table 1). It indicated that lncRNA-OBFC2A took part in the toxicity of benzene.

**Table 1:** The correlation analysis between lncRNA-OBFC2A expression and basic blood test

Correlation coefficient	LncRNA-OBFC2A	WBC	NEUT	RBC	HGB	PLT
LncRNA-OBFC2A	1	**-0.006**	0.002	-0.125*	-0.104*	-0.165*
WBC		1	0.86*	0.205*	0.165*	0.353*
NEUT			1	0.174*	0.128*	0.25*
cRBC				1	0.692*	0.126*
HGB					1	0.037
PLT						1

*Statistically significant correlation between two variables, P<0.05.

Correlation analysis was performed to evaluate the correlation between lncRNA-OBFC2A expression and basic blood test indicators. We used partial correlation analysis to evaluate the correlation between lncRNA-OBFC2A and WBC, NEUT, RBC, HGB and PLT. *Statistically significant correlation between two variables, P<0.05.

### Cell proliferation-related genes expression markedly changed after benzene exposure

**Figure 2:**
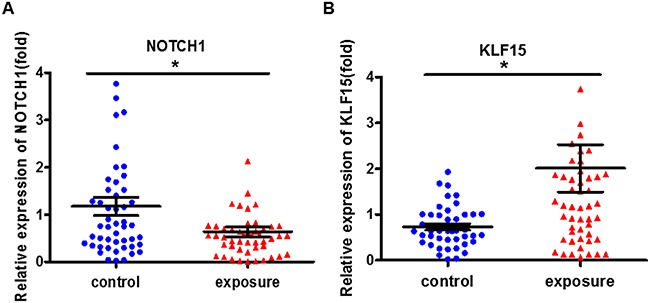
The cell proliferation-related genes expression markedly changed in benzene-exposed workers The expression of NOTCH1 **(A)** and KLF15 **(B)** were analyzed by qRT-PCR. *P<0.05, compared with control. Data are expressed as mean ± S.D.; N=50.

Subsequently, we analyzed the expression of the cell proliferation-related genes NOTCH1 and KLF15 in benzene-exposed workers. As shown in Figure 2, compared with the control, the expression of NOTCH1 decreased in benzene-exposed workers, while the expression of KLF15 increased. It pointed out that expose to benzene markedly changes the cell proliferation-related genes expression.

### LncRNA-OBFC2A was associated with cell proliferation-related genes

**Figure 3:**
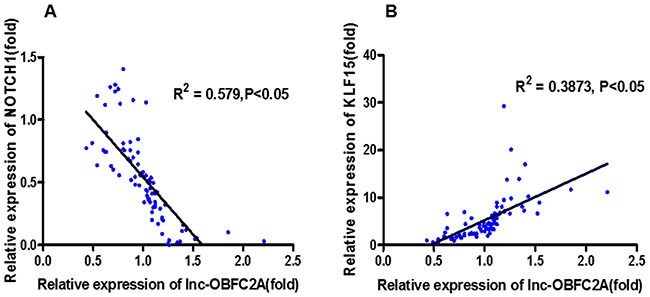
The correlation analysis between lncRNA-OBFC2A expression and cell proliferation-related genes expression *Statistically significant correlation between two variables, P<0.05, n=50.

At the same time, we performed the correlation analysis to explore the role of lncRNA-OBFC2A in benzene-exposed individuals. Results showed that lncRNA-OBFC2A was positively correlated with KLF15 (P<0.05, R^2^=0.3873). While, there was a negative correlation between the lncRNA-OBFC2A expression and NOTCH1 (P<0.05, R^2^=0.579) (Figure 3). All these results suggested that lncRNA-OBFC2A is involved in benzene toxicity by regulating cell proliferation. Next the detailed mechanism was required.

### Cytotoxicity of 1, 4-BQ in AHH-1 cells

**Figure 4:**
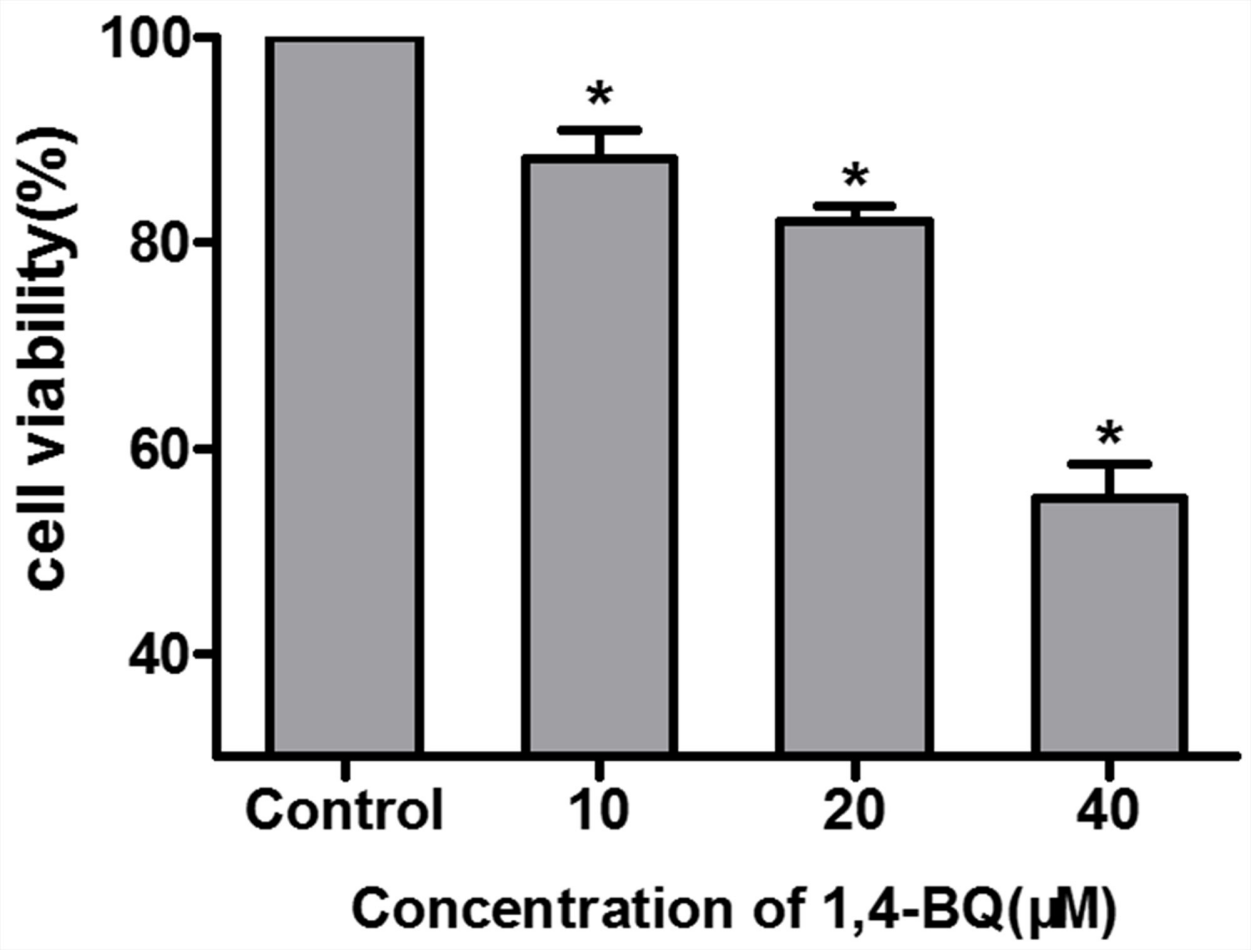
Cytotoxicity of AHH-1 cells induced by 1, 4-BQ Cell viability of AHH-1 cells was measured by an MTT assay after 24 h exposure with different concentrations of 1, 4-BQ. *P<0.05, compared with control group. Data are expressed as means ± S.D. n=3.

Firstly, we chose the metabolites of benzene 1, 4-BQ to evaluate the possible toxicity of benzene in AHH-1 cells and cell viability was determined after exposing cells to 1, 4-BQ (0, 10, 20 and 40 μM) for 24 h. As indicated in Figure 4, viability of AHH-1 cells induced by 1, 4-BQ was significantly lower than that of control. The viability of 20 μM group was 82.11% while 40 μM group was 55.12%. Our results suggested that 1, 4-BQ induced cytotoxicity in a dose-dependent manner.

### Cell proliferation was inhibited by 1, 4-BQ

**Figure 5:**
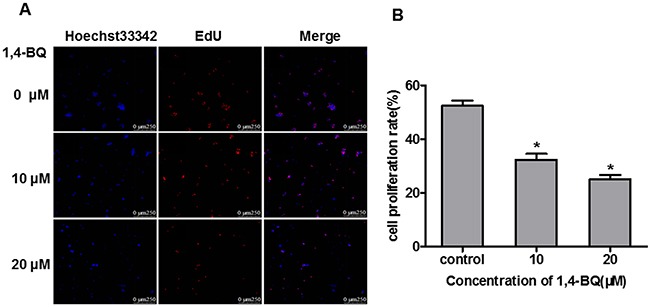
1, 4-BQ dose-dependently inhibited the proliferation of AHH-1 cells **(A)** The blue fluorescence indicates the nucleus of living cells which were dyed by hoechst33342; The red fluorescence indicates the DNA of cells are being copied and purple is the overlap between the two (The nucleus of living cells are stained blue, and the proliferating cells are stained red by EdU); **(B)** Cell proliferation rate (%) = Number of proliferating cells / Number of living cells*100%. Results indicated that 1, 4-BQ dose-dependently inhibited the proliferation of AHH-1 cells. *P<0.05, compared with control. Data are expressed as means ± S.D. n=3.

EdU experiments is a kind of rapid and accurate assessment methods of cell proliferation *in vitro*. Therefore, we performed EdU experiments to study the effect of 1, 4-BQ on cell proliferation. As shown in Figure 5A, the fluorescence intensity of EdU in the experimental group was significantly stronger than that in the control group. Results in Figure 5B showed that the proliferation rate of experimental group was significantly lower than the control group, suggesting that 1, 4-BQ dose-dependently inhibited the proliferation of AHH-1 cells.

### 1, 4-BQ changed cell proliferation-related genes level

**Figure 6:**
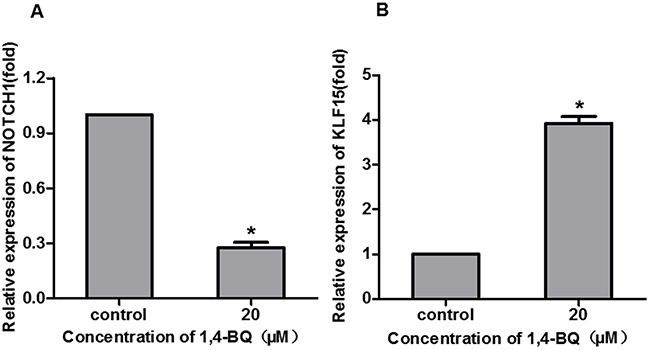
1, 4-BQ changed the proliferation-related gene expression The expression of NOTCH1 **(A)** and KLF15 **(B)** were analyzed by qRT-PCR. *P<0.05, compared with control. Data are expressed as mean ± S.D.; n=3.

According to the results of EdU, we found that 1, 4-BQ markedly inhibited the proliferation of AHH-1 cells. To further investigate the effect of 1, 4-BQ on the expression of NOTCH1 and KLF15, we detected it *in vitro*. As shown in Figure 6, compared with the control group, the expression of NOTCH1 decreased, while the expression of KLF15 increased, which was consistent with the results from population.

### LncRNA-OBFC2A expression in AHH-1 cells induced by 1, 4-BQ

**Figure 7:**
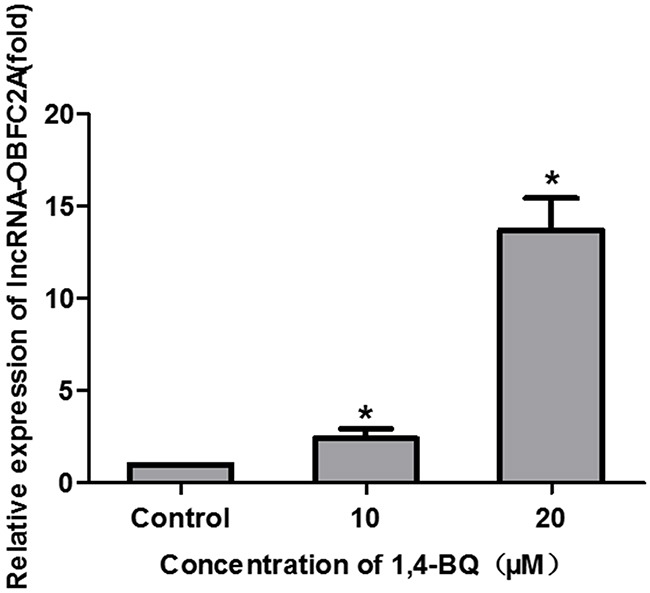
1, 4-BQ up-regulated the expression of lncRNA-OBFC2A in AHH-1 cells The expression of lncRNA-OBFC2A in AHH-1 cells was detected by qRT-PCR. *P<0.05, compared with control. Data are expressed as mean ± S.D.; n=3.

Our previous research has shown that the expression of lncRNA-OBFC2A significantly increased in the peripheral blood of chronic benzene poisoning patients [28]. In this study, we also detected the expression of lncRNA-OBFC2A in AHH-1 cells treated by 1, 4-BQ. Just as revealed in Figure 7, the expression of lncRNA-OBFC2A in experimental group was significantly higher than that of the control group. It is consistent with our preceding results from the peripheral blood of workers exposed to benzene, which evidenced that lncRNA-OBFC2A plays a key role in the toxicity of benzene.

### Down-regulation of lncRNA-OBFC2A was achieved through lentivirus vector transfection

**Figure 8:**
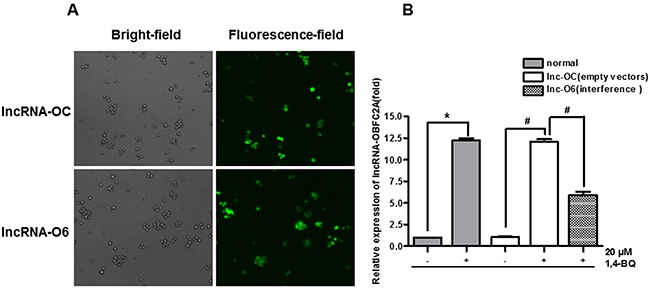
Construct and verify the lentiviral interference model of lncRNA-OBFC2A **(A)** AHH-1 cells were transfected by lentivirus vectors with lncRNA-OBFC2A or empty lentiviral vectors for 72 hours. **(B)** The expression of lncRNA-OBFC2A in AHH-1 cells was detected by qRT-PCR. *p<0.05, compared with normal-, ^#^p<0.05, compared with lnc-OC-. Data are expressed as mean ± SD; n=3. Notes: lnc-OC stands for empty lentiviral vectors group; lnc-O6 stands for lncRNA-OBFC2A interference group; lnc-OC+ stands for the empty lentiviral vectors group with incubation by 1,4-BQ; lnc-O6+ stands for the interference group with incubation by 1,4-BQ.

In order to carry out the functional study of lncRNA-OBFC2A, AHH-1 cells were transfected by lentivirus vectors with lncRNA-OBFC2A or empty lentiviral vectors for 72 h. Lentivirus with green fluorescent protein can identify the successful transfection. When the transfection efficiency was up to 80%, the transfected cells could be used for the following experiments. In addition, ‘lnc-OC’ stands for the group of empty lentiviral vectors, however, ‘lnc-O6’ stands for the lncRNA-OBFC2A interference group. And use ‘+/−’ symbol to represent whether to treat by 1, 4-BQ. As shown in Figure 8A, the ratio of green fluorescent protein expression was up to 80%. Then, the expression of lncRNA-OBFC2A was detected by qRT-PCR. From the result in Figure 8B, there was no significant change between lnc-OC- and normal-, indicating that lentivirus transfection had no effect on lncRNA-OBFC2A expression in AHH-1 cells. While the expression of lncRNA-OBFC2A in lnc-O6+ was lower than that in normal+, suggesting that the expression of lncRNA-OBFC2A was down-regulated in lnc-O6 group. All above results proved that the lentiviral interference model was constructed successfully.

### Interfering lncRNA-OBFC2A alleviated 1, 4-BQ-induced abnormal cell proliferation

**Figure 9:**
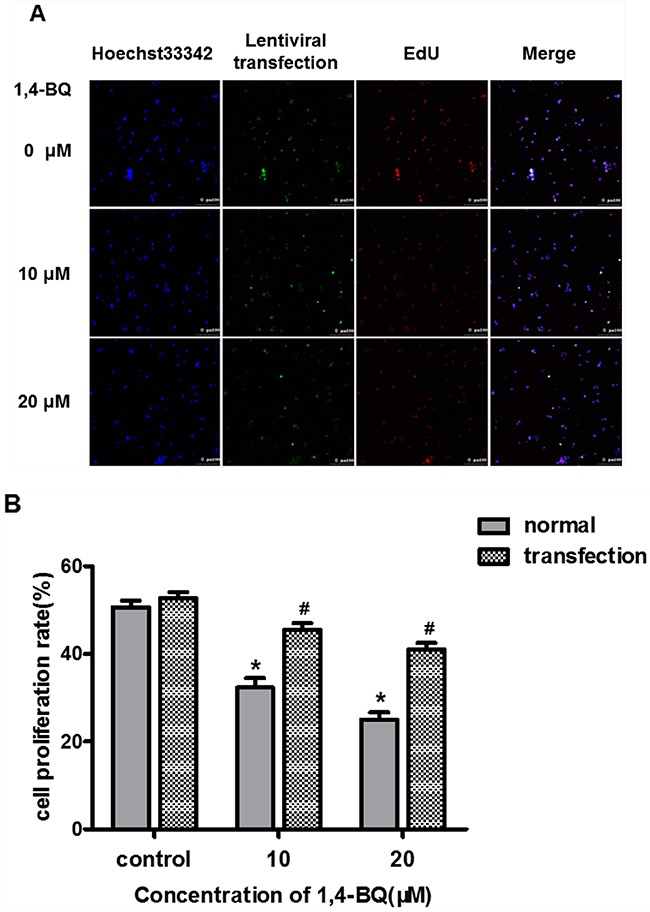
Effect of interfering lncRNA-OBFC2A on cell proliferation induced by 1, 4-BQ **(A)** The blue fluorescence indicates the nucleus of living cells which were dyed by hoechst33342; Green indicates the slow virus transfection fluorescence; The red fluorescence indicates the DNA of cells are being copied and the last is the superposition of the former three; **(B)** The expression of lncRNA-OBFC2A after down-regulating was detected by real-time RCR. *p<0.05, compared with the control group (normal cells). ^#^p<0.05, compare with the transfected-control group. Data are expressed as mean ± SD; n=3.

Subsequently, we treated the transfected AHH-1 cells with 1, 4-BQ for 24 h. The proliferation of AHH-1 cells was detected by EdU technique. Results in Figure 9A showed that the fluorescence intensity of EdU in the experimental group was significantly stronger than that in the control group. From Figure 9B, we observed that compared with the corresponding concentration group before transfection, the cell proliferation rate increased obviously in both 10 μM group and 20 μM group, suggesting that interfering with lncRNA-OBFC2A expression can alleviate the inhibition of 1, 4-BQ-induced cell proliferation in AHH-1 cells.

### The expression of proliferation genes caused by 1, 4-BQ was reversed after interfering lncRNA-OBFC2A

**Figure 10:**
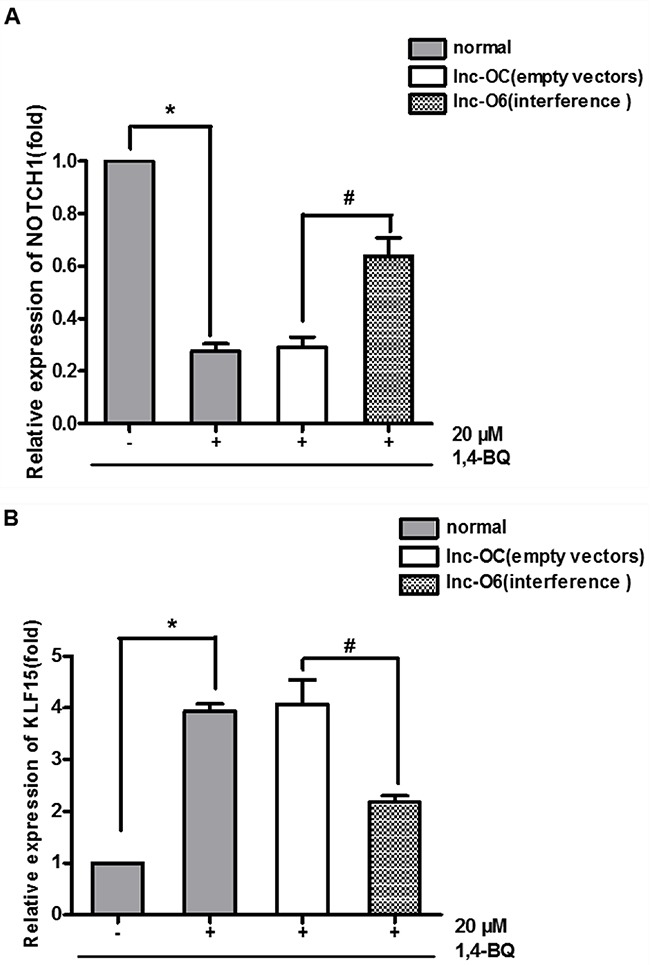
Expression of proliferation genes after interfering the expression of lncRNA-OBFC2A **(A)** Effect of down-regulation of lncRNA-OBFC2A expression on the expression of NOTCH1. The expression of NOTCH1 was analyzed by qRT-PCR. *p<0.05, compared with the normal-, ^#^p<0.05; compared with the lnc-OC+. Data are expressed as mean ± SD; n=3. **(B)** Effect of down-regulation of lncRNA-OBFC2A expression on the expression of proliferation inhibited gene-KLF15. The expression of KLF15 was analyzed by qRT-PCR. *p<0.05, compared with the normal-, #p<0.05; compared with the lnc-OC+. Data are expressed as mean ± SD; n=3.

The early data showed that 1, 4-BQ decreased the expression of NOTCH1, and increased the expression of KLF15. After down-regulating the expression of lncRNA- OBFC2A, we found that compared with normal+, the expression of NOTCH1 in lnc-O6+ increased, while the expression of KLF15 decreased (Figure 10). Results also showed that there was no significant difference between the lnc-OC+ and normal+ exposed group, indicating that the lentiviral vector had no effect on the expression of cell proliferation-related genes. Therefore, lncRNA-OBFC2A inhibits cell proliferation by regulating the expression of proliferation-related genes in AHH-1 cells, which suggests that benzene metabolite decreases cell proliferation via lncRNA-OBFC2A-mediated anti-proliferation effect involving NOTCH1 and KLF15.

### Cell location of lncRNA-OBFC2A

**Figure 11:**
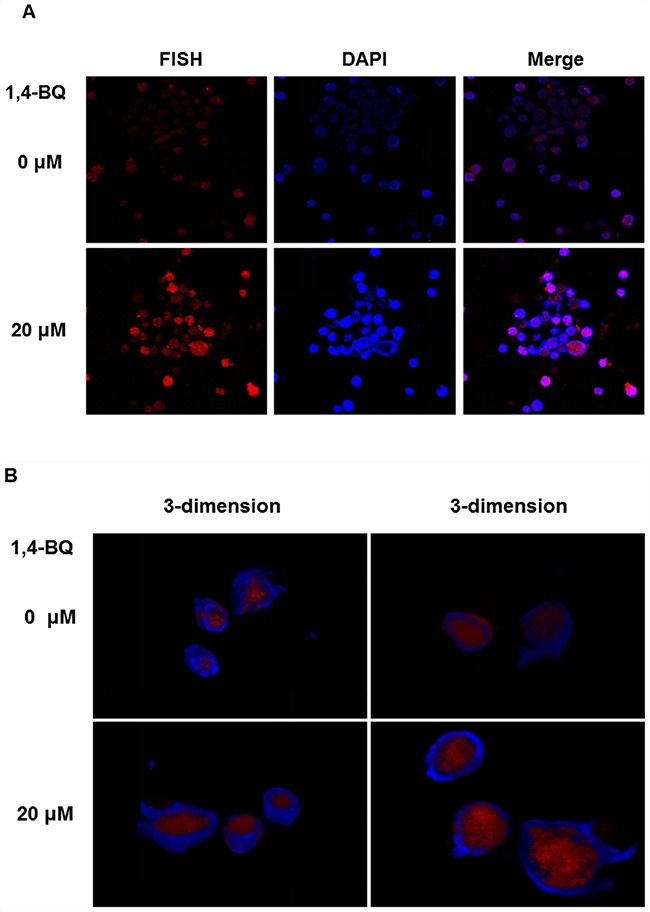
Cell location of lncRNA-OBFC2A The location of lncRNA-OBFC2A in AHH-1 cells was detected by RNA-FISH. **(A)** The plane graph of lncRNA-OBFC2A cell location. The red fluorescence indicates lncRNA-OBFC2A; the blue fluorescence indicates the nucleus of living cells which were dyed by DAPI and the purple is the overlap between the two. **(B)** 3-dimension results of lncRNA-OBFC2A cell location. The red fluorescence indicates lncRNA-OBFC2A; the blue fluorescence indicates the nucleus of living cells which were dyed by DAPI.

These results indicated that lncRNA-OBFC2A participated in the process of cell proliferation by regulating the expression of NOTCH1 and KLF15. In this process, where does lncRNA-OBFC2A play a role? The location of lncRNA-OBFC2A in AHH-1 cells was detected by RNA-FISH after exposure to 20 μM 1, 4-BQ for 24 h. The cell specific probe fluorescence intensity can represent the localization and quantity of lncRNA-OBFC2A. As shown in Figure 11A, the probe fluorescence intensity of lncRNA-OBFC2A in cell nuclei significantly elevated in 1, 4-BQ group. In addition, 3-dimension results of a laser confocal microscopy showed that lncRNA-OBFC2A was indeed localized in the nucleus to take effect (Figure 11B).

## DISCUSSION

Although the toxicity of benzene has been widely concerned, the toxic mechanism of benzene is not yet clear. In recent years, more and more documents indicate lncRNA may be a potential marker of many diseases. Growing studies have shown that many lncRNA molecules are associated with the leukemia [23]. Our previous research has found that lncRNA-OBFC2A was closely associated with chronic benzene poisoning [26]. However, the underlying mechanisms of lncRNA-OBFC2A involved in benzene toxicity has not been clear. Limited studies reveal that lncRNA plays a vital role in cell proliferation. In our study, the results showed that lncRNA-OBFC2A increased in benzene-exposed workers and there was a close correlation between lncRNA-OBFC2A and cell proliferation related-genes. In order to investigate whether lncRNA-OBFC2A participate in the toxicity of benzene through regulating cell proliferation. We used the lentiviral interference model and RNA-FISH assay in AHH-1 cells to explore the role of lncRNA-OBFC2A in benzene toxicity. Our data firstly reported that lncRNA-OBFC2A participated the inhibition of cell proliferation induced by 1, 4-BQ through trans-locating to the nucleus to modulate the expression of NOTCH1 and KLF15. LncRNA-OBFC2A may be a potential biomarker for benzene toxicity.

Cell proliferation is an important life characteristic of living organisms, which including a series process of cell division as DNA replication, RNA transcription and protein synthesis of the complex reaction and DNA replication in nuclear is one of the most important part in the whole process [34].

Ethynyl-2’-deoxyuridine incorporation assay (EdU) is one of the most accurate methods for cell proliferation detection and it can be completed in a few minutes without the DNA denatured [35]. In this study, we investigated the effect of 1, 4-BQ on cell proliferation in AHH-1 cells by EdU. Results showed that 1, 4-BQ dose-dependently inhibited cell proliferation. Moreover, many studies pointed that 1, 4-BQ inhibited cell proliferation. 1, 4-BQ inhibited the colony forming of hBM-HSCs and the cell proliferation of hBM-MSCs *in vitro* [36]. In addition, the cell proliferation of wild-type K562 cells was inhibited after exposing to 1, 4-BQ [37]. All these results are in agreement with our study.

While there are numerous other genes that control cell proliferation, NOTCH1 and KLF15 were very mature and classical genes in cell proliferation studies especially in the area of tumor investigation. The Notch1 signaling pathway regulates many fundamental processes essential for normal development such as the control of cell differentiation, survival, proliferation, and angiogenesis [38]. And it plays a key role in tumor progression of several human cancers [39]. Jinhuang Chen et al. found that PDGF-D positively regulated the expression of Notch1 in CRC cells. Moreover, restoration of Notch1 rescued the inhibition of cell proliferation, migration, and invasion in SW480-shPDGF-D cells [40]. Besides, Konishi J et al. showed that inhibition of Notch signaling by a γ-secretase inhibitor suppresses the growth of non-small cell lung cancer (NSCLC) [41]. Accumulating evidence suggests that Notch signaling plays a critical role in the development of several types of cancer, functioning as a tumor promoter [44–52]. NOTCH1 has been shown to be a gene that promotes cell proliferation [53, 54]. Knocking-down NOTCH1 inhibited cell proliferation of ICC cells and glioma cells [55–57].

However, KLF15 restrains cell proliferation. KLF15 is a transcription factor that is involved in various biological processes, including cellular proliferation, differentiation and death. In addition, KLF15 has recently been implicated in the development of several human malignancies. The results of Yoda T et al. indicated that nuclear KLF15 expression suppresses breast cancer cell proliferation at least partially through p21 up-regulation and subsequent cell cycle arrest [42]. In the human breast cancer-derived cell line T47D, KLF15 has been reported to decrease estrogen-dependent cell proliferation [43]. KLF15 inhibits the proliferation of mesangial cells [44]. Besides, KLF15 suppresses cell proliferation of mesangial cells under high glucose [45] and it negatively regulates estrogen-induced epithelial cell proliferation by inhibition of DNA replication [43]. In our study, we tested the expression of NOTCH1 and KLF15 to explore the cell proliferation of AHH-1 cells induced by 1, 4-BQ. Results showed that 1, 4-BQ decreased the expression of NOTCH1 and increased the KLF15 in AHH-1 cell lines, which is consistent with our results of benzene-exposed population.

In order to explore the role of lncRNA-OBFC2A in benzene toxicity, we detected the expression of lncRNA-OBFC2A *in vitro*, and results showed 1, 4-BQ obviously increased the expression of lncRNA-OBFC2A, which is consistent with our population results. Afterwards, we successfully constructed a lentivirus model of interfering the expression of lncRNA-OBFC2A. Lentivirus vector is a kind of recombine retrovirus vector, as an important gene transfer tool, it is used in the field of gene therapy and other cellular and molecular biology [46,47]. In order to interfere the expression of lncRNA-OBFC2A, we used lentivirus vector to transfect AHH-1 cells. Lnc-O6 stands for the lncRNA-OBFC2A interference group, however, lnc-OC stands for the group of empty lentiviral vectors. Further, transfection efficiency was verified by detecting the expression of lncRNA-OBFC2A. After interfering lncRNA-OBFC2A, the cell proliferation inhibition and the expression of proliferation-related genes induced by 1, 4-BQ were reversed. A series of studies report that the lncRNA has an effect on cell proliferation. LncRNA AK126698 inhibits proliferation of non-small cell lung cancer cells by targeting Frizzled-8 and suppressing Wnt/β-catenin [48]. LncRNA-MEG3 inhibits cell proliferation of endometrial carcinoma by repressing Notch signaling [49]. The high expression of lnc-HOTAIR can promote the proliferation, migration and invasion of tumor cells, while the unlimited proliferation of cells is closely related to the occurrence and development of cancer [50]. LncRNA-SNHG7 promotes the proliferation of lung cancer cells by enhancing the FAIM2 expression [51]. Our results indicated lncRNA-OBFC2A affected cell proliferation by regulating the expression of proliferation-related genes in AHH-1 cells.

Above results indicated that lncRNA-OBFC2A participated in the process of cell proliferation by regulating the expression of NOTCH1 and KLF15. In order to make it clear that where does lncRNA-OBFC2A work, we measured it by Fluorescence in situ Hybridization (FISH) [52]. FISH was widely used in conjunction with banded chromosome analysis, and as a stand-alone technique for the detection of genomic alterations in neoplastic disorders [53]. Besides, FISH can be used to detect the specific existence sequence of DNA or RNA in cells and for genetic markers, chromosomal aberrations, chromosomal location of genes [54]. The cell specific probe fluorescence intensity can represent the localization and quantity of the goal RNA. Our results showed that the fluorescence intensity of experimental group was higher than that of control, indicating that lncRNA-OBFC2A was localized in the nucleus to take effect.

Taken together, lncRNA-OBFC2A regulates the expression of proliferation- related genes NOTCH1 and KLF15 in cell nuclei, which affects the cell proliferation inhibition induced by 1, 4-BQ. And LncRNA-OBFC2A can be seemed as a promising biomarker for benzene toxicity in the early stage of benzene exposure.

## MATERIALS AND METHODS

### Characters of study design

In this study, 50 workers were exposed to 3.50±1.60 mg/m^3^ concentration of air benzene and 50 controls who were exposed to 0.06±0.01 mg/m^3^ air benzene [33]. Each participant was required to fill a questionnaire including demographic, life-style and occupational information, such as age, gender, medications history, smoking history, drinking history and family history of health status. The study was approved by the Committees for Ethical Review of Research involving Human Subjects of Capital Medical University. All participants provided the written informed consents.

### Routine blood detection

Blood samples were collected from workers for hematology analysis. We used an automated blood analyzer (Brand) to detect the routine blood indicators including White blood cell (WBC), neutrophil (NEUT), red blood cell (RBC), platelet (PLT) and haemoglobin (HGB).

### RNA isolation and qRT-PCR

Total RNA was extracted with Reagent kit (TIANDZ, China) according to the manufacturer's instructions. Quantificational real-time polymerase chain reaction for lncRNA-OBFC2A with specific primers from Sangon Biotech (Shanghai, People's Republic of China) was performed using Revert Aid First Strand Kit (Thermo Fisher Scientific, USA) and KAPA SYBR FAST Universal qPCR kit (KAPA, US) according to the manufacturer's protocol. β-actin was used as an endogenous control to normalize mRNA levels. The sequences of primers were as follows: lncRNA-OBFC2A: forward: 5′GTTGGTGTGCGGAGTGGTT3′; reverse: 5′GCAGAAAGCCGTTAGTCAGG3′; NOTCH1: forward: 5′CAATGAGTTC

CAGTGCGAGT3′; reverse: 5′GTAAGTGTTG GGTCCGTCCA3′; KLF15: forward: 5′TACACCAA AAGCAGCCACCT3′; reverse: 5′TCTTCTCGCACACA GGACAC3′; β-actin: forward: 5′TGAGACCTTCA ACACCCCAG3′; reverse: 5′GCCATCTCTTGCTC GAAGTC3’. Real-time reverse transcription-polymerize chain reaction was performed on Bio-Rad (CFX96™ optics module). Each sample was repeated three times. Data were analyzed by comparing cycle threshold values.

### Cell culture and 1, 4-BQ treatment

The human normal lymphocyte line (AHH-1) was given as a gift by the National Institute for Radiological Protection, China CDC (Chinese Center for Medical Response to Radiation Emergency). The cells were cultured at 37°C in 5% CO_2_ humidified environment, and maintained in RPMI Medium 1640 basic (RPMI-1640) (Gibco, USA) supplemented with 10% donor equine serum (HyClone, USA), 100 U/mL penicillin, and 100 μg/mL streptomycin. For tests, the cells were seeded into 6-well plates at a density of 1×10^6^ cells/mL and allowed to attach for 24 h, then treated with 1, 4-BQ (0, 10, 20 and 40 μM ) for 24 h. The equivalent volume of RPMI-1640 without 1, 4-BQ was used as control group.

### MTT assay

The cytotoxicity of 1, 4-BQ was detected by 3-(4,5-dimethyl-2-thiazolyl)-2,5-dipheny l-2-H-tetrazolium bromide (MTT) assay. About 1×10^6^ cells/mL AHH-1 cells were seeded into a 96-well plate, then cells were incubated with various concentrations (0, 10, 20 and 40 μM) of 1, 4-BQ at 37°C for 24h. A microplate reader (Thermo Multiscan MK3; Thermo Fisher Scientific, Waltham, MA, USA) was used to measure the absorbance of formazan at 492 nm.

### EdU assay

Ethynyl-2’-deoxyuridine incorporation assay was performed using an EdU Apollo DNA *in vitro* kit (KeyGEN, China) following the manufacturer's instructions. Briefly, single cell suspension was prepared from the cells in the logarithmic growth phase. And about 1×10^6^ cells/mL AHH-1 cells were seeded into a 96-well plate with various concentrations (0, 10 and 20 μM) of 1, 4-BQ at 37 °C for 24 h. Then the cells were suspended liquid moved to 2 mL EP tube, 1500 r/min, 5 min centrifuged supernatant, each hole added 1mL 1×EdU working solution, and incubated at room temperature for 2 h, then discard the working solution, each hole incubated with 0.5 mL reaction mixture for 30 min at room temperature in the dark. The samples were washed before being observed under fluorescence microscopy. Laser confocal microscope was used to detect the proliferation.

### Lentivirus infection

Lentivirus vectors were constructed in Genechem (Shanghai, People's Republic of China). These lentivirus vectors contain a target gene (lnc-O6) or empty lentiviral vectors (lnc-OC) and a green fluorescent protein marker. Single cell suspension was prepared from the cells in the logarithmic growth phase. To generate stable clones, 1×10^5^ cells/mLAHH-1 cells were seeded into a 6-well plate. Afterwards, 5×10^6^ transducing units of lentivirus with 500 μL of enhanced infection solution and polybrene (5μg/mL) were mixed to infect AHH-1 cells. And we observed the situation of green fluorescent protein marker after 72 h for detecting infection efficiency by the fluorescence microscope (OLYMPUS, Japan).

### RNA fluorescence in situ hybridization assay

The location of lncRNA-OBFC2A in AHH-1 cells was detected by RNA fluorescence in situ Hybridization (RNA-FISH). Cells were fixed in 4% poly formaldehyde for 30 min at room temperature, then the sample was dehydrated with increasing concentrations of ethanol (70%, 85% and 100%). Coverslips were incubated and hybridized overnight with the RNA-FISH probes in a humidified environment at 37 °C and dyed the nucleus with DAPI. The fluorescence was measured by a laser scanning confocal microscope (Leica, Germany).

### Statistical analysis

Data were expressed as mean ± S.D. of three independent experiments, and significance was determined by using one-way analysis of variance (ANOVA) followed by least significant difference (LSD) test to compare the differences between groups. In all cases, P<0.05 was considered to be statistically significant.
